# Low-cost electrochemical detection of l-tyrosine using an rGO–Cu modified pencil graphite electrode and its surface orientation on a Ag electrode using an *ex situ* spectroelectrochemical method

**DOI:** 10.1039/d0ra04015k

**Published:** 2020-06-16

**Authors:** C. Kavitha, K. Bramhaiah, Neena S. John

**Affiliations:** Department of Physics, Center for Advanced Materials Research, B.M.S. Institute of Technology & MGMT, Affiliated to VTU Avalahalli, Yelahanka Bengaluru-560064 Karnataka India gkavitha21@bmsit.in gkavitha21@gmail.com +080-65369468; Centre for Nano and Soft Matter Sciences Jalahalli Bengaluru-560013 India

## Abstract

A low cost reduced graphene oxide–copper hybrid nano thin-film modified Pencil Graphite Electrode has been employed to detect the l-tyrosine enantiomer. The free-standing rGO–Cu hybrid nano-thin film was prepared by a simple one-step liquid–liquid interface method. Electrochemical Cyclic Voltammetry, Differential Pulse Voltammetry, pH-dependent and scan rate dependent studies on bare PGE, Cu, rGO, and rGO–Cu for l-tyrosine have been explained in detail. The rGO–Cu modified PGE based biosensor exhibits good detection of l-tyrosine. The linear range detection limit was estimated to be 1 × 10^−7^ M. The calculated sensitivity is 0.4 μA ppm^−1^ mm^2^. This electroactive biosensor is easily fabricated and controlled and is cost-effective. The surface orientation of l-tyrosine on the Ag electrode at a particular potential and its comparison with vibrational DFT calculations have been studied for the first time.

## Abstract 2

A low cost reduced graphene oxide–copper hybrid nano thin-film modified pencil graphite electrode has been employed to detect the l-tyrosine enantiomer.

## Graphical abstract

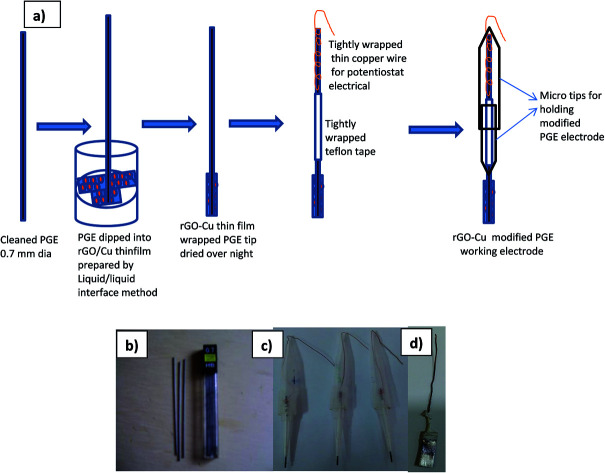

## 1. Introduction

The success of new materials lies in their high performance, earth-abundance, cost-effectiveness, eco-friendliness, and biocompatibility. A recent challenge is the detection of biomolecules, disease diagnosis, and its early treatment. In this connection, tremendous research work is going on around the world with different types of materials, synthesis routes, detection methods, and so on. Amino acids are building blocks of protein. Tyrosine is one of the small, electroactive, and aromatic amino acids which is used to regulate the signal transduction process in proteins. In human beings, tyrosine is an important precursor of thyroid hormones, dopamine, adrenaline, and so on, which is used to establish and maintain balanced nutrition.^1,2^ Tyrosine deficiency causes diseases like hypothyroidism, hypochondria, and dementia. In order to detect tyrosine, several techniques like high performance liquid chromatography (HPLC),^3^ electrophoresis,^4,5^ fluorimetry,^6^ chromatography-mass spectrometry^7,8^ are used. However, these methods involve extensive sample preparation and time-consuming measurements. Hence, there is a need to develop a simple, rapid, and stable method that is low cost. Electrochemical detection is the best method and is easy to operate.^9^ Carbon-based materials are frequently used as biosensors. In particular, graphene oxide and its nanoparticle hybrids are recent rising stars due to their unique properties like high surface area, enhanced electrical and thermal conductivity, high mechanical strength, and optical transparency. A multi-walled carbon nanotube (MWCNT) electrode was used for voltammetric responses of tyrosine.^10^ MWCNT–ionic liquid hybrid electrodes were used for tyrosine detection.^11^ It has been reported that a glassy carbon electrode modified with reduced graphene oxide shows a strong electroactive surface than any other carbon nanomaterials.^12^ This shows the graphitic structure is highly important for efficient biosensing. Though the above-mentioned electrodes perform quick and accurate detection, the preparation and modification of these electrodes are complex.

In this article, we have developed a reduced graphene oxide/copper hybrid nano-thin film using a simple liquid/liquid interface method. The electrodes used here are low-cost pencil graphite electrodes (PGE). The as-prepared hybrid nano-thin films are transferred on to PGE and used for detection. Secondly, in order to study the surface orientation of l-tyrosine, we have chosen *ex situ* potential dependent electrochemical surface-enhanced Raman spectroscopy (EC-SERS) method. EC-SERS is an emerging tool to understand the architecture of the adsorbed species at a molecular level, their conformational changes upon adsorption and the identification of functional group that is directly involved in the bonding on the surface is of particular importance in many more biological applications.^13–16^ For example, J. Bukowska *et al.* shows potential dependent adsorption of enantiomeric and racemic forms of methionine on silver electrode surface probed by EC-SERS.^17^ Density Functional Theory (DFT) method is a non-invasive tool for quantum mechanical modeling. In many cases, the results of DFT calculations for solid-state systems agree quite satisfactorily with experimental data. We have used Gaussian 09 DFT calculations to calculate vibrational modes of l-tyrosine on the Ag substrate.^18^

## 2. Materials and methods

### 2.1 Synthesis

**Fig. 1:**
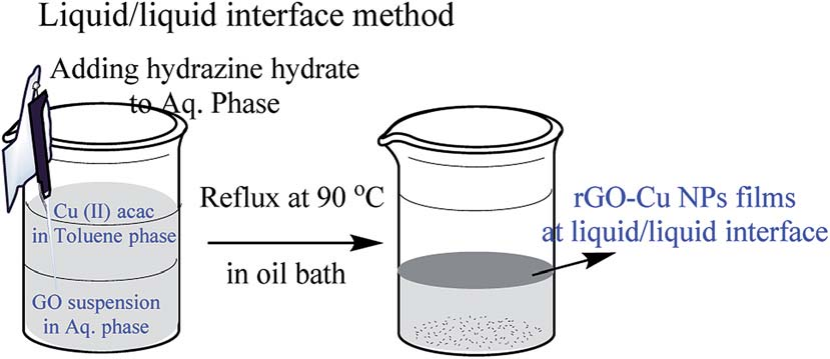
Schematic representation of the liquid/liquid interface method.

The following chemicals toluene (HPLC grade, 99.8%), hydrazine hydrate (reagent grade 50–60%), graphite flakes (99% carbon basis, −325 mesh particle size), Cu(ii) acac (≥99.9% trace metals basis) were purchased from Sigma-Aldrich and used as received. In a typical synthesis, 5 mL (toluene) of 1.5 mM solution of Cu(ii) acac mixed with 20 mL of toluene served as the upper phase, and 25 mL of Milli Q water containing 5 mg of graphene oxide as the bottom phase. 50 μL of NH_2_NH_2_·H_2_O (hydrazine hydrate) was added to the aqueous phase with minimum disturbance and the system was heated in an oil bath at 90 °C for 1 h. After 1 hour, an ultra-thin film of rGO–Cu NPs observed at the liquid/liquid interface.^19,20^ The schematic representation of the liquid/liquid interface method is shown in Fig. 1.

Modified Hummers method was used to prepare exfoliated graphene oxide (GO) from graphite particles (300 mesh, Alfa Aesar). GO was purified, sonicated and a standard solution of 0.5 mg mL^−1^ GO in deionized water was prepared for further use. For the preparation of rGO films, a standard solution of GO is prepared and 25 mL of 0.5 mg mL^−1^ of GO in 100 mL beaker is taken followed by the addition of 10–20 μL of 80% THPC (in water) and heated at 100 °C for 1 h. A thin film of rGO is formed at the air/water interface.^21^ This thin film is collected on the desired substrate for further characterization and applications.

### 2.2 Working electrode preparation

The working electrode has been prepared by taking 0.7 mm dia PGE. Initially, the graphite rods were thoroughly cleaned before the use. PGEs were immersed in dilute HNO_3_ solution for 15 minutes followed by washing with Milli-Q water. A plastic microtip served as a holder into which the graphite rod wrapped tightly with Teflon tape was inserted.

**Fig. 2:**
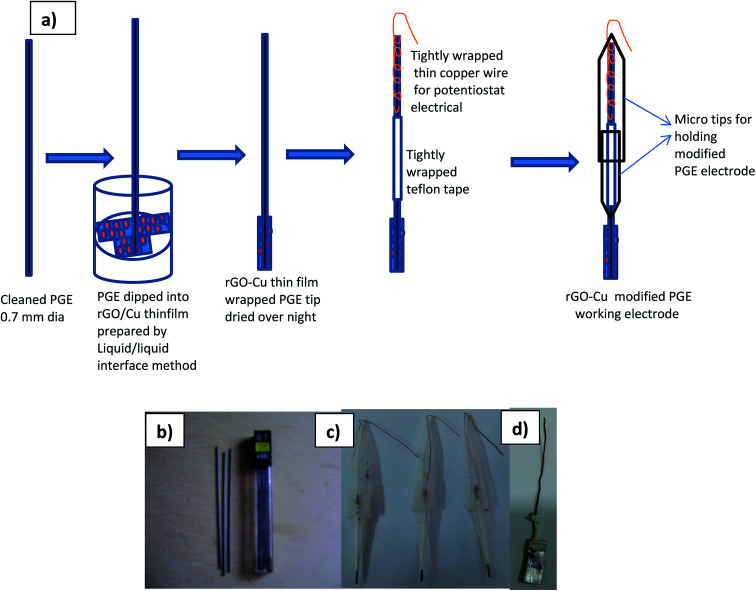
Schematic fabrication of (a) rGO/Cu modified PGE, (b) 0.7 mm pencil graphite needle, (c) as prepared rGO/Cu modified PGE, (d) as prepared Ag electrode.

Keeping 2 mm of the rod tip exposed at one end of the holder. In order to make an electrical connection with the potentiostat, a copper wire was attached to the other end of the graphite rod and passed through another plastic 1 mL micro tip. The as-synthesized rGO–Cu NPs hybrid films prepared by liquid/liquid interface method were collected on PGE tip and dried overnight. The same way the roughened silver electrode was used for SERS studies on l-tyrosine. The schematic fabrication and photograph of as prepared rGO/Cu modified PGE, silver working electrodes are as shown in Fig. 2a–d.

### 2.3 Characterization

The surface morphology and compositions of as-synthesized films are examined by field-emission scanning electron microscopy (FESEM) and energy-dispersive X-ray spectroscopy (EDS) by Nova NanoSEM600, FEI and MIRA 3 LMH, TESCAN. For SEM imaging, the thin films were collected on doped Si substrates washed and dried under argon gas. The operating voltage of FESEM was 10 kV in a high vacuum equipped with a through-lens detector. UV-visible absorption spectra were recorded using a Perkin Elmer Lambda20 spectrophotometer. X-ray diffraction (XRD) studies of as-synthesized samples were done using the Rigaku Smart Lab Diffractometer equipped with parallel beam optics and Cu-K_α_ radiation (*λ* = 1.54 Å, 40 kV, 30 mA) was incident at the grazing angle 0.3°. CH instruments electrochemical system (660 E series) was employed for all electrochemical measurements like cyclic voltammetry (CV), and differential pulse voltammetry (DPV). A conventional three-electrode system was used with the modified PGE as the working electrode, a standard Ag/AgCl (sat. KCl) electrode as the reference electrode, and a platinum electrode as a counter electrode. A standard stock solution of l-tyrosine (1 ppm, 10 ppm, and 100 ppm) is prepared with Milli-Q water. For the preparation of various pH buffer solutions, we have used standard procedures. 10 mL of 0.1 M phosphate buffers with various pH such as 1, 2, 3, 5.8, 7 and 8 were prepared from the standard solution of 0.1 M KCl, HCl, K_3_HPO_4_, and NaOH. In this study, we have used 1 mL of 100 ppm l-tyrosine in 0.1 M phosphate buffer solution for electrochemical studies.

### 2.4 Analyte under study: l-tyrosine

**Fig. 3:**
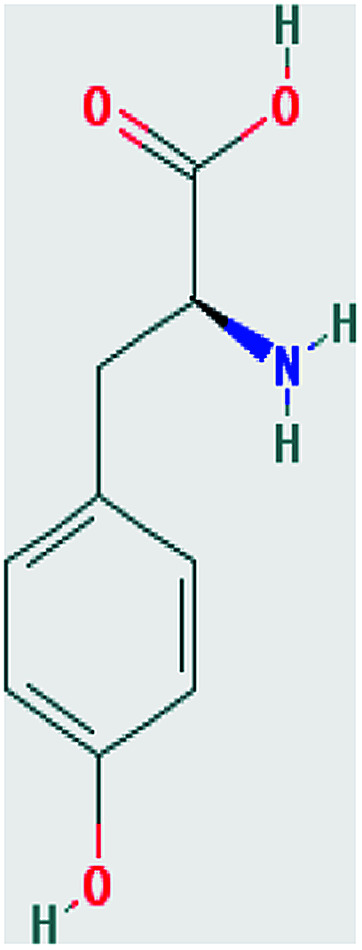
Chemical structure of l-tyrosine.

Tyrosine or 4-hydroxyphenylalanine is one of the 20 standard amino acids that are used by cells to synthesize proteins. It is a non-essential amino acid with a polar side group. This can be detected by electrochemical methods because it is an electrochemically active compound. The main advantages of electrochemical methods are that they are low cost, fast, simple, and convenient to operate. The chemical formula of l-tyrosine is C_9_H_11_NO_3_, and the chemical structure is shown in Fig. 3. Its molar mass is 181.19 g mol^−1^. It has 0.453 g/100 mL solubility in water.

### 2.5 *Ex situ* EC-SERS

The electrochemical cell for SERS measurements consists of a silver strip as a working electrode with a purity of 99.99%, an Ag/AgCl (sat. KCl) as a reference electrode, and a Pt wire as a counter electrode. A CH Instrument Model 660 E series was used for controlling the electrode potential. The silver electrode was cleaned thoroughly with emery paper prior to each experiment and then roughened with an electrochemical oxidation–reduction cycle (ORC) in 1.0 M KCl solution by applying 3 cycles from 0 V to −0.5 V and back to 0.1 V at a sweep rate of 50 mV s^−1^. At the end of the roughening process, the Ag electrode was kept at −500 mV for 30 s in the same solution. The silver electrode was removed at an open circuit potential, rinsed with distilled water and then transferred to the solution of (0.01 M l-tyrosine) + (0.1 M KCl) in the electrochemical cell for electrochemical adsorption of the molecule on the Ag electrode at 0.15 V. After adsorption, the Ag electrode was taken out and thoroughly cleaned with distilled water and SERS was performed on a dried silver electrode. The solutions were prepared with MilliQ water. The l-tyrosine was supplied by the SISCO Research lab and was used as received. Experimental Raman spectra of l-tyrosine with different excitation wavelengths, 532 and 633 nm have been recorded. The spectra were recorded using a Horiba Xplora Raman instrument with 25% of laser power for 1 s accumulation time. In each experiment, a freshly roughened Ag electrode and fresh l-tyrosine solution were used.

### 2.6 Theoretical calculations

Density functional theory (DFT) is a quantum mechanical modeling method used in physics and chemistry to investigate the electronic structure of particular atoms, molecules, and the condensed phases. DFT was used in many areas; it is interesting for their great agreement with the experimental results in calculating vibrational frequency. Density functional theory (DFT) vibrational calculations on the surface were carried out with the Gaussian 09 software package using the combination of the B3PW91 exchange–correlation functional along with the Lanl2DZ basis set. All system structures were optimized and the Raman frequencies for the optimized structures were calculated. Raman frequencies for the optimized structures of solid l-tyrosine were calculated by B3LYP/6-31G (d). A uniform scaling factor of 0.9613 has been adopted for all the computed frequencies corresponding to the vibrational normal modes.^18^ The density functional calculation using B3LYP was chosen among the different DFT methods due to its good performance in molecular structure and vibrational modes determinations.^22^ In the case of vibrational calculations of l-tyrosine on the Ag surface, two models of l-tyrosine/silver system structures on the substrate were optimized. The vibrational frequencies for the optimized structures were calculated with DFT-B3PW91/Lanl2dz based on two models.

## 3. Results & discussions

**Fig. 4:**
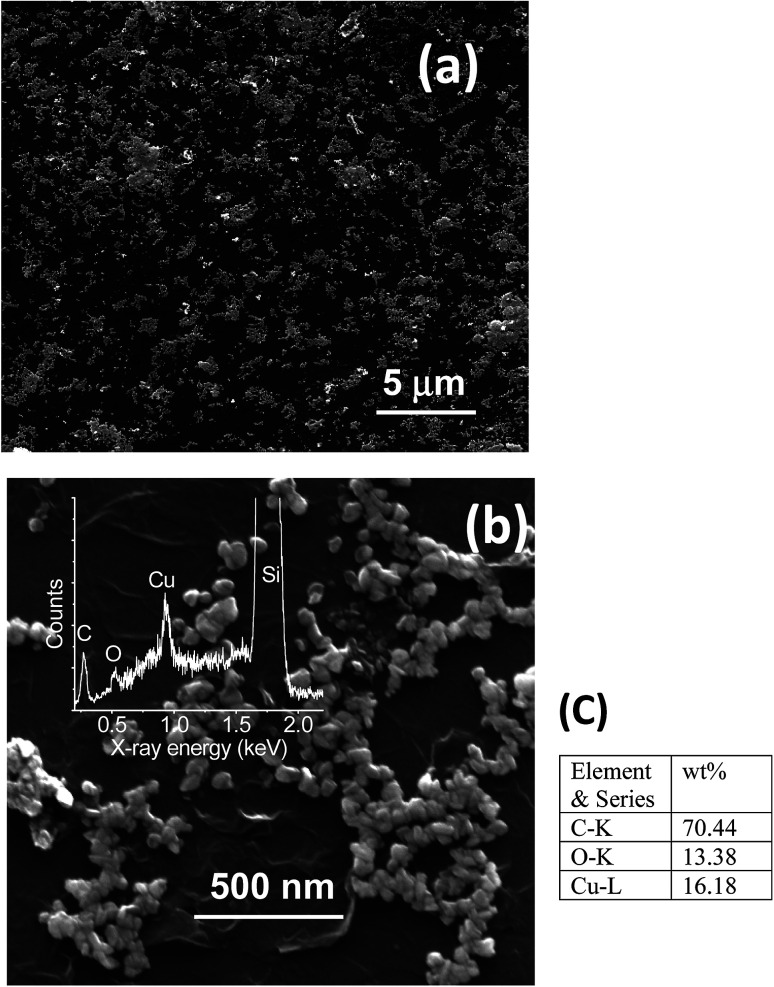
FESEM images of rGO–Cu NPs hybrid film prepared at a liquid/liquid interface (a) low magnification image (b) high magnification image along with EDS spectra. (c) EDS elemental weight percentage.

Fabrication of hybrid thin films *via* self-assembly employing a liquid/liquid interface approach is an attractive method for the generation of hybrid materials. In the present case, we fabricated an rGO–Cu NPs hybrid film employing a liquid/liquid interface approach. The approach involves the introduction of Cu precursor in the toluene phase over the GO suspension in aqueous phase followed by the addition of hydrazine hydrate as a reducing agent. As reaction progresses, the *in situ* reduction of Cu ions and GO leads to the formation of a free-standing, rGO–Cu NPs thin film at a liquid/liquid interface. These hybrid films can be transferred to any desired substrate for further characterization and demonstration of their applications. The schematic representation of the synthesis process of the rGO–Cu NPs hybrid thin-film employing a liquid/liquid interface approach is shown in Fig. 1. The surface morphology of as-synthesized rGO–Cu NPs hybrid films has been studied by FESEM and displayed in Fig. 4.

We have shown a low magnification and high magnification FESEM images along with EDS data. From Fig. 4a, one can be seen that the Cu NPs are distributed over the rGO layers. These Cu NPs are aggregated in a few areas and nonspherical in nature, which can be seen from the magnified FESEM image (Fig. 4b). The elemental analysis measurements have been achieved employing EDS (inset of Fig. 4b) and exhibit the C, O, and Cu elements from rGO and Cu. The EDS elemental weight percentage have been shown in Fig. 4c.

**Fig. 5:**
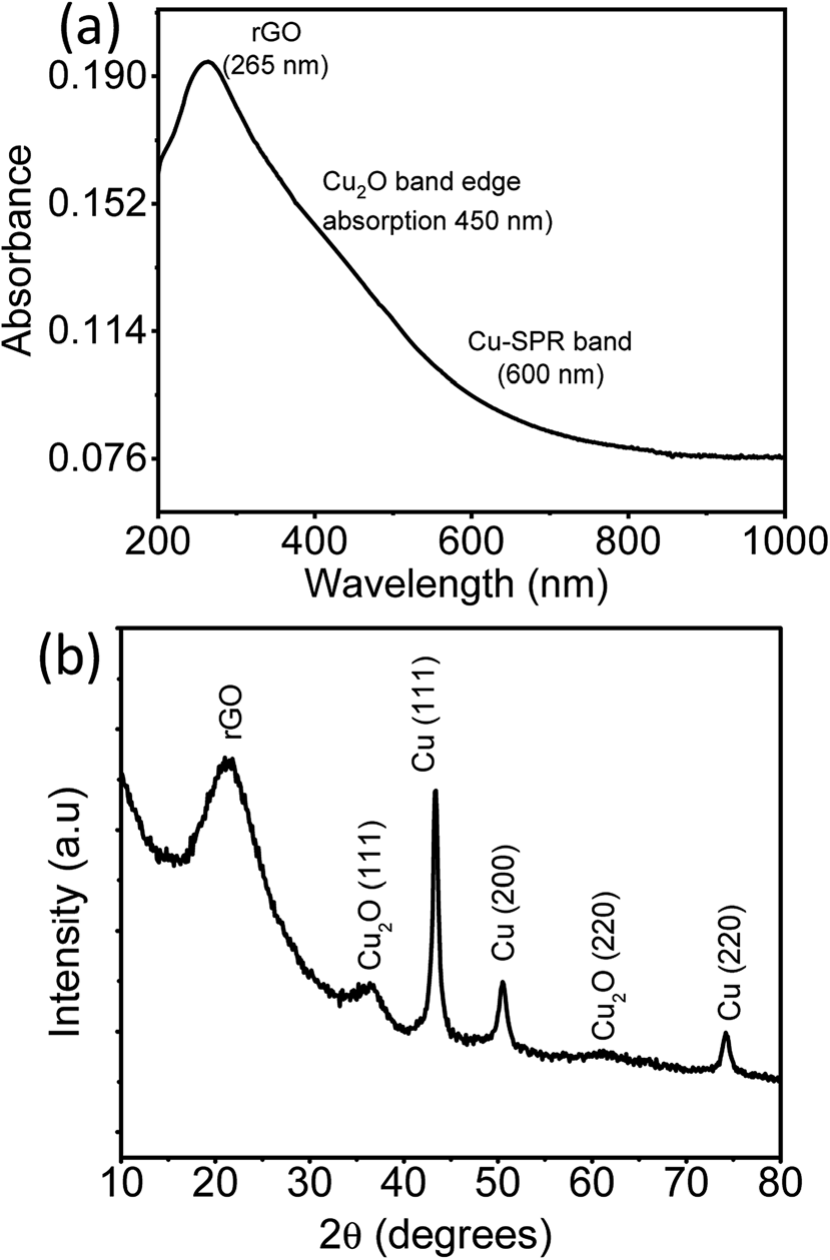
(a) UV-Visible spectra (b) XRD pattern of rGO–Cu NPs hybrid film obtained by a liquid/liquid interface method.

The absorption spectra and the X-ray diffractogram of as prepared rGO–Cu hybrid thin film prepared by a liquid/liquid interface method are shown in Fig. 5a and b. It is clear from Fig. 5a that the peak at 265 nm is attributed to extended conjugation in the basal plane carbon network of reduced graphene oxide.^21^ The surface plasmon resonance (SPR) band of Cu nanoparticles observed at 600 nm along with band-edge absorption of Cu_2_O at 450 nm.^23,24^ The crystalline phase of an as-prepared rGO–Cu hybrid substrate was probed by X-ray diffraction (XRD) in the range 10–80°. The broad peak at 21.3° is attributed to exfoliated rGO sheets. The sharp Cu peaks are observed at 43.4°, 50.4° and 74.1° correspond to Cu (111), Cu (200), and Cu (220) crystalline planes of face-centered cubic copper (JCPDF: 03-1018), respectively. A few small peaks due to cubic Cu_2_O are observed at 36.5° and 61.2° corresponding to Cu_2_O (111), Cu_2_O (220) planes, respectively.

### 3.1 Electrochemical sensing of l-tyrosine

**Fig. 6:**
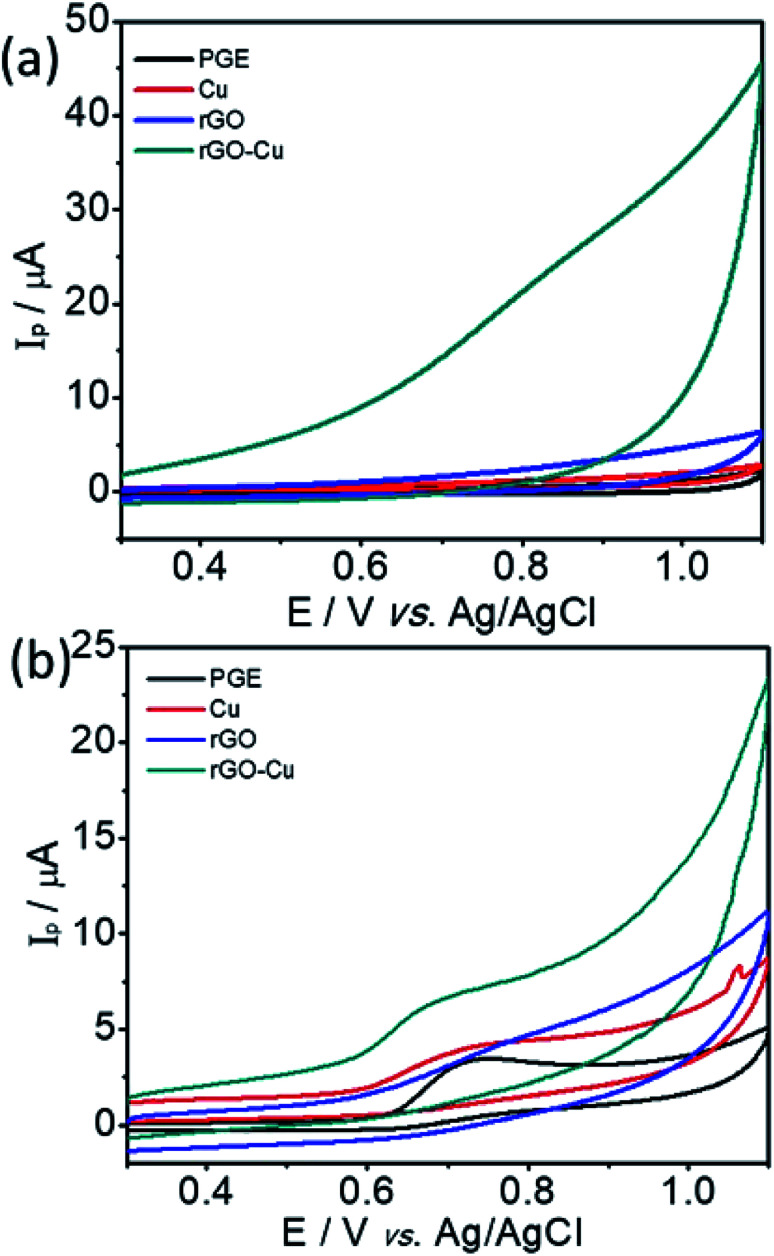
CV curves in the absence (a) and presence (b) of 100 ppm of l-tyrosine at various electrodes including bare PGE, and PGE modified with Cu, rGO, rGO–Cu films obtained by liquid/liquid interface method in 0.1 M phosphate buffer solution (pH 7.0) with a scan rate of 50 mV s^−1^.

The electrochemical sensing of l-tyrosine at various electrodes such as bare PGE, Cu NPs film, rGO and rGO–Cu NPs hybrid film-coated over PGE are studied by cyclic voltammetry (CV) measurements in 0.1 M phosphate buffer solution (PBS) containing 100 ppm concentration of l-tyrosine at a scan rate of 50 mV s^−1^ and the CV results are depicted in Fig. 6. Prior to the l-tyrosine measurements, CV scans for all modified electrodes are taken in blank PBS solution at pH 7 and no redox peaks were observed (Fig. 6a). The rGO–Cu NPs hybrid film exhibits higher current than the other electrodes including bare PGE, Cu NPs, and rGO film. From the Fig. 6b, it is clear that there is only one anodic peak is observed at all modified electrodes such as bare PGE, Cu NPs, rGO and rGO–Cu NPs hybrid film when the potential is scanned from 300 mV to 1100 mV with the addition of 100 ppm of l-tyrosine, which indicates that the oxidation reaction is irreversible. The observed oxidation peak potentials (*E*_p_) of l-tyrosine at the working electrodes such as bare PGE, Cu NPs, rGO, and rGO–Cu NPs films are 730 mV, 710 mV, 790 mV, and 670 mV, respectively. In the case of rGO–Cu NPs hybrid film case, the oxidation peak potential (*E*_p_) of l-tyrosine shifted to the negative potentials along with higher currents indicating that the rGO–Cu NPs hybrid films exhibiting greater electrocatalysis for the oxidation of l-tyrosine. The reasons for enhanced electrocatalysis of rGO–Cu NPs hybrid films for the oxidation of follows: (1) the rGO–Cu NPs hybrid film can improve the electron transfer rate greatly because of the superior conductivity of rGO layers (2) l-tyrosine has the aromatic and various functional groups including –COOH, –NH_2_ and –OH, so there might be π–π interactions and hydrogen bonding between the rGO layer and l-tyrosine (3) higher surface area of the rGO–Cu NPs hybrid films compared with other electrode materials, resulting in higher adsorption of analyte molecules leading to higher current values, which can be observed from the Fig. 6b. All these synergistic functions of the rGO–Cu NPs hybrid film make a contribution to superior electrocatalytic performance.

**Fig. 7:**
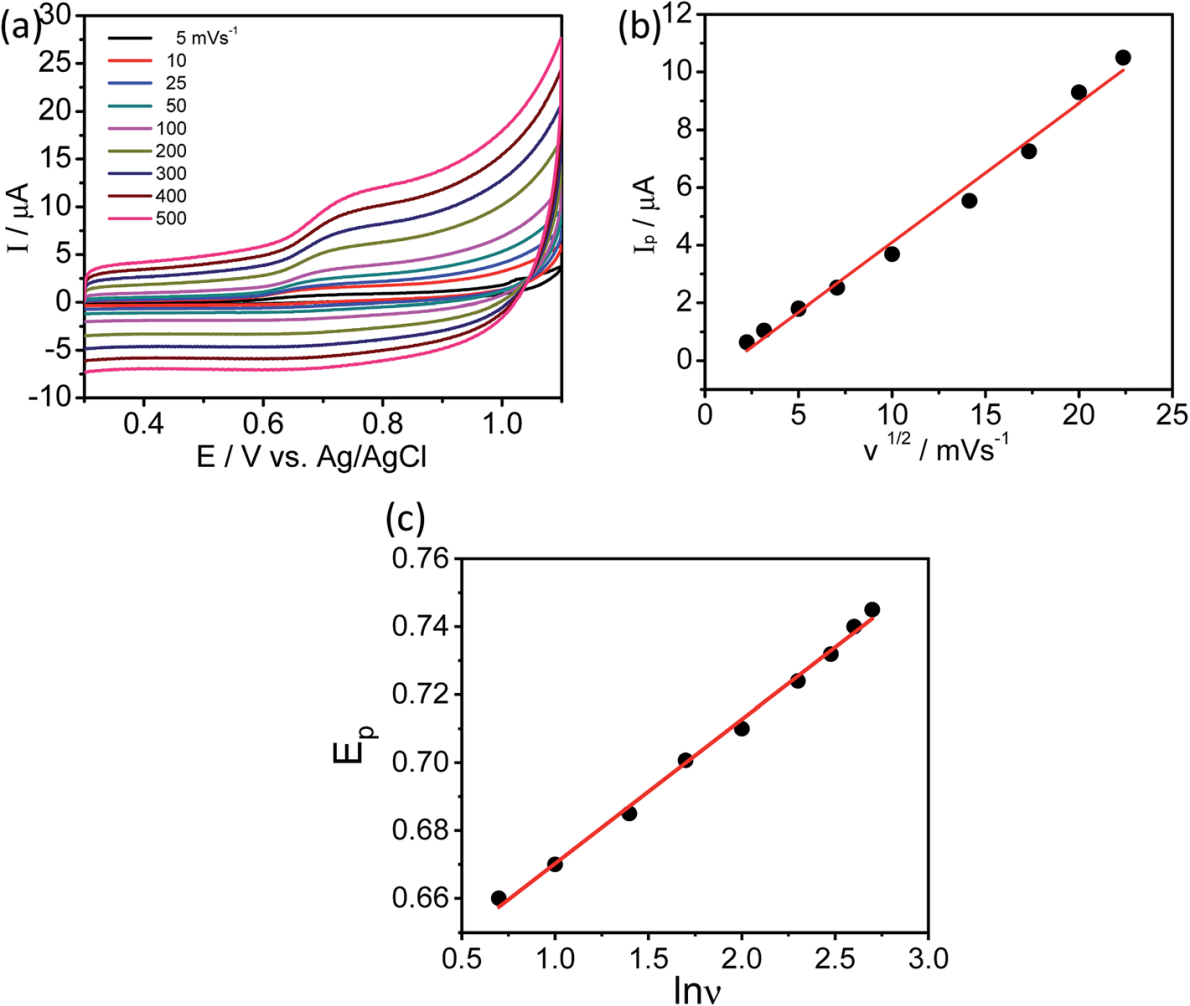
(a) CV of l-tyrosine (100 ppm) in 0.1 M phosphate buffer solution at rGO–Cu NPs hybrid film-coated over PGE with different scan rates. (b) Plot of peak current (*I*_p_) *vs.* square root of scan rate (*ν*^1/2^) and (c) plot of peak potentials (*E*_p_) *vs.* log of scan rate (ln *ν*).

The effect of scan rate on the oxidation of l-tyrosine at the rGO–Cu NPs hybrid thin-film collected over PGE electrode with different scan rates ranging from 5 to 500 mV s^−1^ in 0.1 M phosphate buffer solution (pH-7) containing 100 ppm concentration of l-tyrosine response is studied and CV curves are displayed in Fig. 7a. The obtained results disclose that the current values and anodic peak currents (*I*_p_) gradually increase with the increasing square root of the scan rate (*ν*^1/2^) which is indicative of diffusion control (Fig. 7b). The higher current values are observed with increasing scan rate due to the electric double layer mechanism and fast charge/discharge process along with higher ohmic resistance at higher scan rates. A good linear relation was observed between the peak current and scan rate. The observed linear regression equation to be *I*_p_ = 4.82*ν*^1/2^ (mV s^−1^) − 7.35 (*R*^2^ = 0.987). These results recommend that the electrochemical oxidation reaction of l-tyrosine on the rGO/Cu NPs hybrid film-coated on PGE is a surface controlled electrochemical process. The anodic peak potential (*E*_p_) gets shifted to more positive potential values with increasing scan rate for the oxidation of l-tyrosine. This is expected for an irreversible process. Moreover, by plotting peak potential (*E*_p_) *vs.* ln *ν*, a straight line is obtained (Fig. 7c). It can be expressed as *E*_p_ = 0.0424 ln *ν* + 0.627 (*R*^2^ = 0.99). For an irreversible electrode process, the *E*_pa_ is defined employing the Laviron theory.^25^
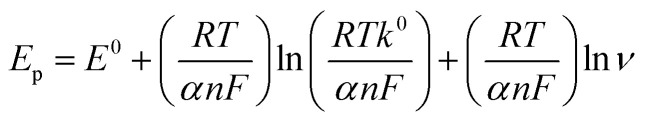
where *E*_p_ is the anodic peak potential (V), *E*^0^ is the standard peak potential (V), *α* is the transfer coefficient (0.5) and *k*^0^ is the standard heterogeneous rate constant of the reaction, *n* is the number of electrons involved in the reaction, *F* is the Faraday constant and *ν* is the scan rate. From the above equation, one can easily calculate the number of electrons transferred. The slope of *E*_p_*vs.* ln *ν* = (2.303*RT*)/*nαF*, where *α* value is around 0.5 for the irreversible system and the calculated value for *n* is 2. Therefore, the electrochemical oxidation of l-tyrosine at the rGO–Cu NPs hybrid film over the PGE is a two-electron transfer reaction.

**Fig. 8:**
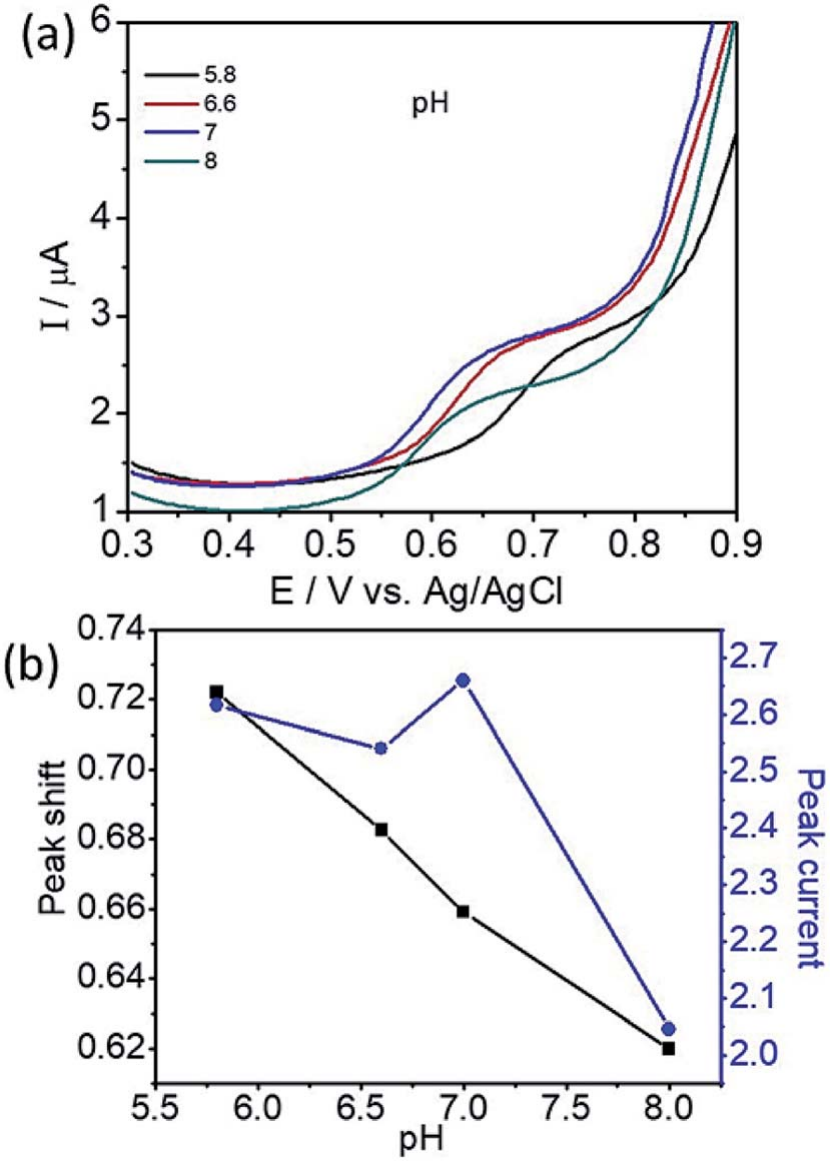
(a) DPV curves of l-tyrosine at various pH and (b) plot for the variation of peak potential (*E*_p_) and peak current (*I*_p_) with the pH.

**Fig. 9:**
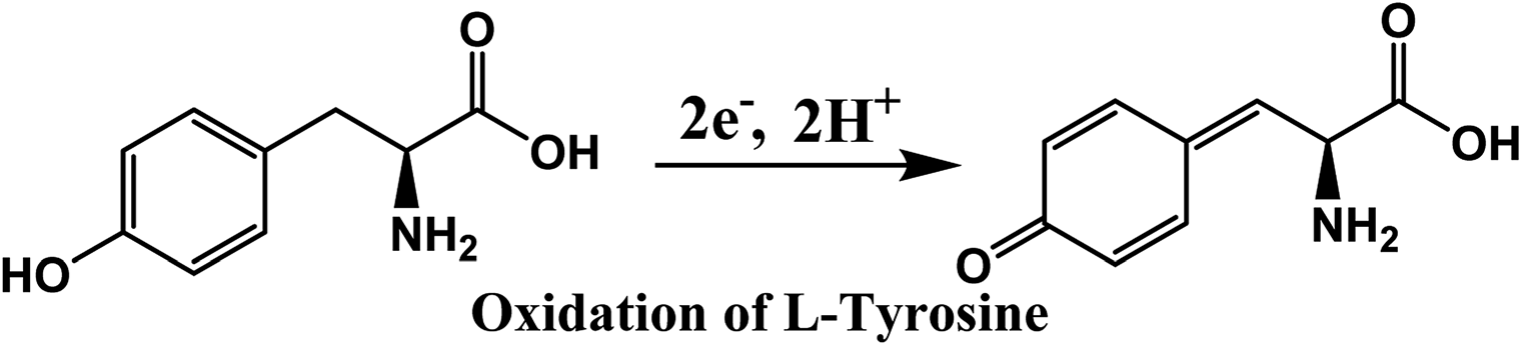
Electrochemical oxidation mechanism of l-tyrosine over the rGO–Cu NPs hybrid film-coated on the PGE electrode.

To determine the effect of solution pH over the electrooxidation of l-tyrosine, we conducted experiments in the range of pH from 5.8 to 8. The DPV curves of l-tyrosine oxidation over the rGO–Cu NPs hybrid film-coated on PGE is shown in Fig. 8. The oxidation peak current of l-tyrosine increases with increasing the pH and reached a maximum value at a pH of 7. After that, the peak current decreases with increasing the pH. Therefore, a pH of 7 was chosen for the subsequent experiments. The oxidation peak potentials of l-tyrosine shifted negatively with the increment of solution pH (Fig. 8b), which indicates that the proton is involved in the electro-oxidation process of l-tyrosine on the rGO–Cu NPs hybrid film-coated over the PGE electrode. The peak potentials of l-tyrosine followed the linear regression equations with pH: *E*_pa_ (V) = −0.0465pH + 0.9899 (*R*^2^ = 0.99). The value of the slope was −0.0465 pH^−1^, which is close to the theoretical value of −0.058 pH^−1^.^26^ From the above results, it can be deduced that the proton number is equal to the number of transferred electrons. Thus, the irreversible oxidation of l-tyrosine on the rGO–Cu NPs hybrid film is proven the participation of two protons (2H^+^) and two electrons process. It means that not only electrons but also protons are released from the l-tyrosine during the electrochemical oxidation process. The schematic of the electrochemical oxidation mechanism of l-tyrosine on the rGO–Cu NPs hybrid film-coated over the PGE is displayed in the given Fig. 9.

### 3.2 Electrochemical determination of l-tyrosine using DPV

**Fig. 10:**
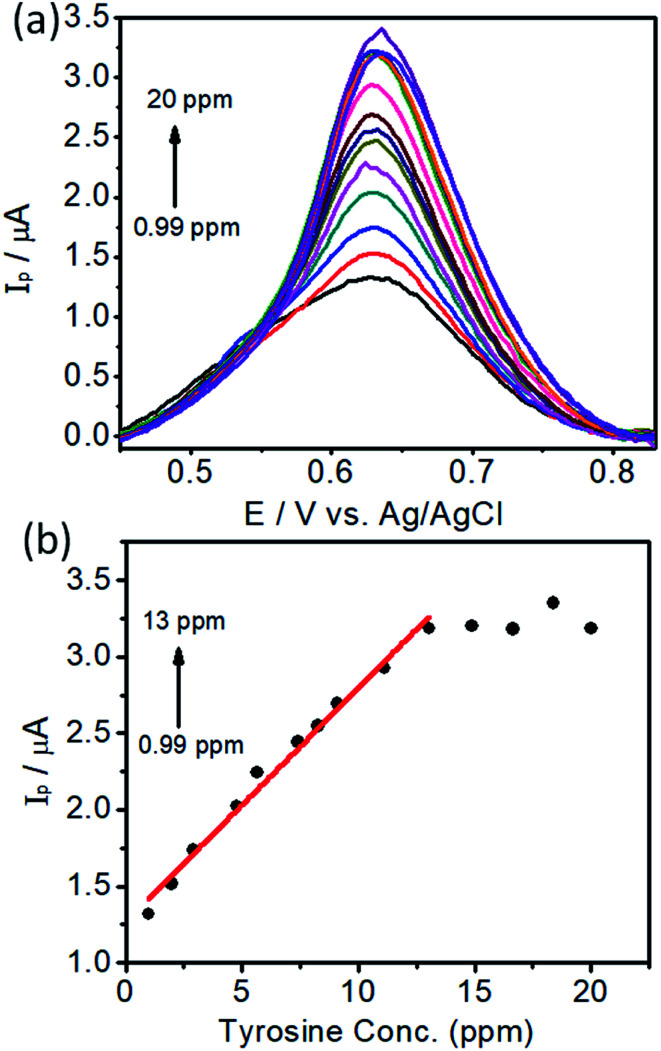
(a) Differential Pulse Voltammetry (DPV) curves of different concentrations of l-tyrosine on rGO–Cu NP film-coated PGE and (b) peak currents (*I*_p_) *versus* concentration of l-tyrosine.

In order to develop an electrochemical method for the detection of l-tyrosine, we employed a DPV approach. Under the optimal conditions, the DPV curves were obtained for the l-tyrosine solution with various concentrations on the rGO–Cu NPs hybrid films coated over PGE and the results are depicted in Fig. 10. It can be seen that the well-defined oxidation peaks were observed and the peak current increased with the increase of l-tyrosine concentration. l-Tyrosine concentration *versus* peak current is plotted and shown in Fig. 10b. From Fig. 10b, one can observe that the peak current increases linearly with an increase in l-tyrosine concentration range from 0.99–13 ppm, which can be a calibration curve for the detection of l-tyrosine as an analyte. Furthermore, with increasing the concentration of l-tyrosine, the peak current (*I*_p_) gets saturated and the sensitivity decreases. The linear calibration curve has been described by the linear regression equation, *I*_p_ = (0.153)*C* + 1.266 (*R*^2^ = 0.99). The detection limit was estimated to be 1 × 10^−7^ M. The calculated sensitivity can be 0.4 μA ppm^−1^ mm^2^. The sensitivity is a ratio of slope in Fig. 10b and the electrode area of 0.38 mm^2^. The preeminent performance of rGO–Cu NPs hybrid film-coated over PGE electrode for the electrochemical detection of l-tyrosine can be attributed to the synergic effect of the rGO–Cu NPs hybrid, which has a large surface area and good dispersity. The l-tyrosine molecules having the phenyl ring along with various functional groups such as –COOH, and –NH_2_ groups, which interact with the rGO through π–π interaction and hydrogen bonding and electrostatic interactions. Furthermore, rGO is a good adsorbent for the adsorption of analyte molecules. The oxidation of l-tyrosine molecules is facilitated by the electron transfer between the l-tyrosine molecules and the rGO–Cu NPs hybrid film.

In the first linear range of lower concentration of l-tyrosine, the number of l-tyrosine molecules is very less. Whenever an anodic current is presented, a single l-tyrosine molecule will be oxidized directly.^27^ Here the slope represents the single l-tyrosine molecules that will be oxidized by electrons. Beyond 13 ppm with more number of l-tyrosine molecules, the peak current is almost saturated. The decrement of the slope can be attributed to the electrocatalytic ability of rGO–Cu on PGE being saturated beyond 13 ppm of l-tyrosine concentration. The detection limit and the linear range of rGO–Cu modified PGE are comparable with the sophisticated glassy carbon electrode (GCE)^28^ and l-serine polymer on GCE.^29^ The synergic effect of rGO–Cu on the PGE electrode and the π–π interaction between them results in a relatively lower detection limit. The above evidence shows that the low-cost rGO–Cu on PGE electrode prepared through a liquid/liquid interface method is very simple to use and more effective in the determination of l-tyrosine levels compared to other sophisticated methods.

### 3.3 *Ex situ* electrochemical SERS measurement

The amino acids are in general accepted as determining the adsorption mechanism of the peptides on to a given metal surface. Therefore, analyzing the SERS signal from adsorbed species allows us to understand the possible ways in which the peptides interact with the surrounding medium and their binding nature on the surface. Because of adsorbate interactions with the metal surfaces, certain bands that are strong in conventional Raman spectra may not be present in the SERS spectra, and *vice versa* (*i.e.* weak bands in ordinary Raman spectra may be clearly observed in SERS spectra). Although interpretation can be difficult, SERS is governed by a surface selection rule that states that vibrations with large tensor components oriented along the vertical axis to the metal surface will be enhanced most. In our report, the SERS spectra have been recorded on electrochemically adsorbed l-tyrosine on to the previously roughened silver electrode at a particular potential. Theoretical DFT-SERS calculations are also done for l-tyrosine. Upon comparison with the experiment, we have proposed the possible orientation of l-tyrosine on the Ag electrode.

**Fig. 11:**
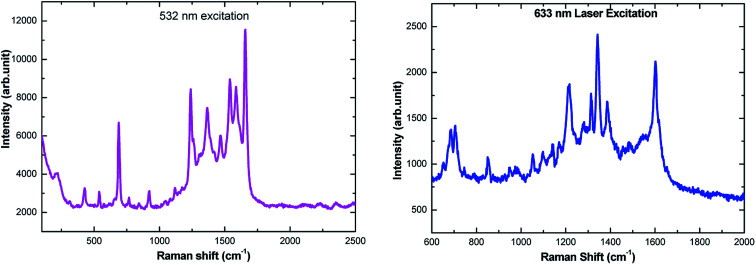
*Ex situ* SERS spectra of 150 μL of 0.01 M l-tyrosine adsorbed on Ag electrode at 0.15 V potential acquired at 532 nm and 633 nm laser excitation.

The Raman spectra of 0.01 M l-tyrosine adsorbed on the roughened Ag electrode at 0.15 V is shown in Fig. 11. The adsorbed electrode was cleaned and dried and two excitation wavelength 532, 633 nm has been used to record Raman spectra. Though we have Raman modes at a higher frequency region (2900–3100 cm^−1^), the fingerprint region is (200–1800 cm^−1^). So we restrict our interpretation in this region only. The detailed SERS Raman bands and their assignments are shown in Table 1.^30–32^

**Table 1:** SERS bands of l-tyrosine and their assignments

532 nm excitation	633 nm excitation	Band assignment
210	—	In-plane C–C deformation
307	—	Wagging of C_1_–C_2_
425	—	In plane deformation of C–O
537	—	Torsion of OH
—	652	COO^−^ wagging
690	684	Out of plane deformation of C–H
	705	Angle deformation of CO_2_
	744	Out of plane deformation of C–H ring
768		Out of plane deformation of C–H ring
	789	Out of plane deformation of C–H ring
848	851	Fermi resonance between ring breath and out-of-plane ring bend overtone CH_2_ rock
920	—	C–COO^−^ stretching
	947	C–COO^−^ stretching
965	979	Stretching of C–N
1052	1053	In-plane deformation of C–H
1084	1098	In-plane deformation of C–H
1124	—	NH_3_ rocking
1140	1143	NH_3_ rocking
1167	1167	NH_3_ rocking
—	1214	NH_3_ rocking
1236	1235	C–O stretching
1276	1283	Aromatic
1310	1312	CH_2_ wagging
1367	1339	In plane angle deformation of OH
1466	1387	C–C stretching
1536	1489	Angle deformation of NH_3_
1589	1545	Angle deformation of NH_3_
1660	1601	Angle deformation of NH_3_

**Fig. 12:**
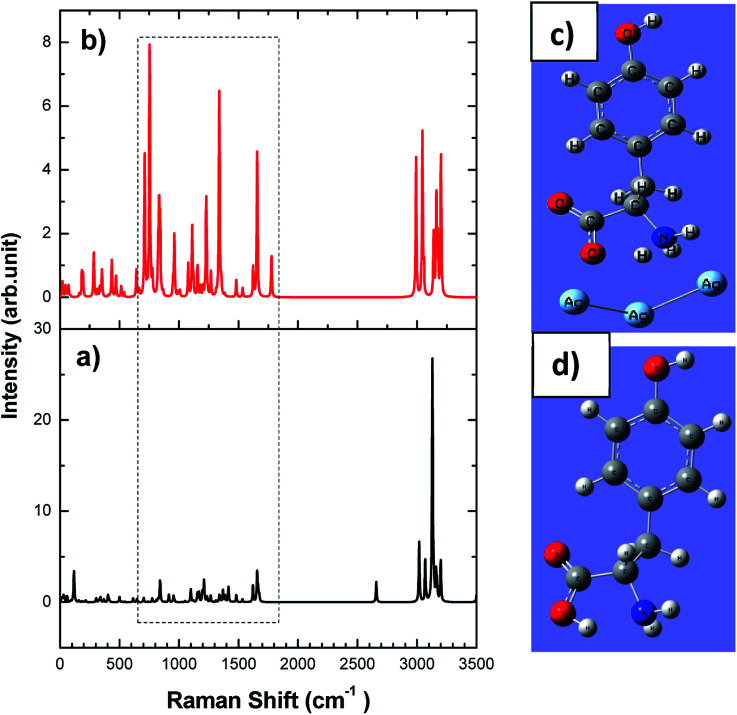
DFT calculation of l-tyrosine (a) solid, (b) electromagnetic adsorption on the Ag surface. The ball and stick model shows the DFT optimized structure of l-tyrosine (c) solid, (d) electromagnetic adsorption on the Ag surface. The dotted rectangular box shows the enhanced Raman bands due to the Ag substrate.

In order to understand the adsorption mechanism of l-tyrosine on the Ag electrode, we have done DFT calculations for solid l-tyrosine and l-tyrosine adsorbed on the Ag substrate with electromagnetic enhancement mechanism. The optimized configuration of l-tyrosine in solid form, and that adsorbed on the Ag electrode and their corresponding theoretical Raman spectra are shown in Fig. 12a–d.

Experimental Raman bands and their mode assignments are shown in Table 1. It is clear from the table that the wavenumber region from 900–1700 cm^−1^ mostly related to vibrations of carboxylate and NH_3_ functional groups which are enhanced due to electrochemical adsorption of l-tyrosine on Ag substrate with 0.15 V of electrode potential. This observation is precisely consistent with the theoretical SERS spectra of electromagnetic mechanism (see Fig. 12b). So it is evident that the carboxylate and NH_3_ functional group of l-tyrosine are nearer to the Ag surface. The optimized configuration of l-tyrosine adsorbed on the Ag surface also shown in Fig. 12c. It is clear from the figure that carboxylate and NH_3_ functional groups are involved in the adsorption of these amino acids on the silver particles. l-Tyrosine adsorbs on silver electrode only through the carboxylate, NH_3_ group with the phenyl ring being perpendicular or slightly tilted to the surface. This has been observed for the first time.

## 4. Conclusions

Electrochemistry plays an important role in electrochemical biosensor and to detect the surface orientation of the molecule. It has been demonstrated that rGO–Cu on the PGE electrode prepared through a liquid/liquid interface method is low cost and very simple to use and more effective in the determination of l-tyrosine levels compared to other sophisticated methods. The excellent performance, good stability, and reproducibility of the substrate together with ease of its fabrication made this an attractive tool to sense different electrochemically active species. Further, the synergic properties of rGO–Cu on PGE can detect effectively l-tyrosine. The detection limit was estimated to be 1 × 10^−7^ M. Potential dependent surface-enhanced Raman spectroscopy is used to probe the surface orientation of l-tyrosine amino acids for the first time. DFT-SERS calculations are consistent with experimental data. The possible orientation of l-tyrosine on the Ag electrode at 0.15 V potential has been proposed. The potential-dependent possible orientation of enantiomers and a racemic mixture of (d, l, dl) tyrosine are under progress in future research.

## Conflicts of interest

There is no conflict to declare.

## Supplementary Material
